# BRCA1 and p53 Tumor Suppressor Molecules in Alzheimer’s Disease

**DOI:** 10.3390/ijms16022879

**Published:** 2015-01-28

**Authors:** Atsuko Nakanishi, Akari Minami, Yasuko Kitagishi, Yasunori Ogura, Satoru Matsuda

**Affiliations:** Department of Food Science and Nutrition, Nara Women’s University, Kita-Uoya Nishimachi, Nara 630-8506, Japan; E-Mails: GAH00635@nifty.com (A.N.); laa_minami@cc.nara-wu.ac.jp (A.M.); y_kitagishi@live.jp (Y.K.); pl0okm9ijn@live.jp (Y.O.)

**Keywords:** BRCA1, p53, Alzheimer’s disease, tumor suppressor, DNA repair, cell signaling, dietary supplementation

## Abstract

Tumor suppressor molecules play a pivotal role in regulating DNA repair, cell proliferation, and cell death, which are also important processes in the pathogenesis of Alzheimer’s disease. Alzheimer’s disease is the most common neurodegenerative disorder, however, the precise molecular events that control the death of neuronal cells are unclear. Recently, a fundamental role for tumor suppressor molecules in regulating neurons in Alzheimer’s disease was highlighted. Generally, onset of neurodegenerative diseases including Alzheimer’s disease may be delayed with use of dietary neuro-protective agents against oxidative stresses. Studies suggest that dietary antioxidants are also beneficial for brain health in reducing disease-risk and in slowing down disease-progression. We summarize research advances in dietary regulation for the treatment of Alzheimer’s disease with a focus on its modulatory roles in BRCA1 and p53 tumor suppressor expression, in support of further therapeutic research in this field.

## 1. Introduction

Alzheimer’s disease (AD) is a progressive degenerative disorder that commonly affects emotional stability and memory domains, and is the most predominant reason of dementia [1,2]. Pathologically, AD is characterized by the damage of neurons and its synapses [3,4]. Although research efforts have provided insights into the biology of AD, the underlying pathways mediating the progressive decline in cognitive function are still poorly understood. However, there are obviously a number of parallels between AD and cancer, including age and other risk factors [5,6]. Nucleic acid damage (DNA damage) susceptible to neurodegeneration and cell death is well-documented in AD and cancer [7,8]. Cells are prepared with the various DNA repair mechanisms to deal with the DNA damage and transduce the signal downward, which provokes a process to inhibit cell cycle progression, and to induce DNA repair [9,10]. The chief DNA damage recognition molecule is ataxia telangiectasia-mutated (ATM), which is a checkpoint kinase that phosphorylates a number of proteins including BRCA1 and p53 in response to the DNA damage, and thus induces a response [11,12]. The ATM protein appears to sense double strand breaks (DSB) during mitosis and/or several other DNA breaks consequent to the damage of free radicals [12]. Accordingly, mutations in the ATM have been associated with increased risk of developing a cancer. It is also well-known that mutations in the *BRCA1* and *p53* tumor suppressor genes comprise a variety of cancers. In general, tumor suppressor molecules play a pivotal role in regulating both cell proliferation and cell death in many cell types. Those may also play an important role in progression of the AD lesion coinciding with changes in the cellular composition. For example, alteration of the tumor suppressor gene p53 function, essential in DNA repair and cell apoptosis, often exacerbates cognate behaviors [13]. Other tumor suppressor proteins such as p21 and p27 activated by BRCA1 are also involved in DNA damage [11,14] as well as in AD [15,16]. Such tumor suppressors play a neuro-protective role in cell survival instead of apoptosis. The p53 protein is a key transcription factor that regulates some signaling pathways involved in the cellular response to genome stresses and DNA damage. Through the stress-induced activation, p53 initiates expression of target genes which protect the genetic reliability of cells. In this way, normal cells show an outstanding balance through the various mechanisms of DNA repair. Consequently, genomic instability is often related to the DNA repair deficiencies [17,18]. Standard DNA repair pathways existing in mammalian cells comprise homologous repair, single strand annealing, non-homologous end joining and so on; these are the different pathways that repair DNA double strand breaks (DSBs). An intricate set of signaling pathways identify the DSBs and mediate either survival via DNA repair or apoptotic cell death [19]. The DNA damaging agents generally used for cancer therapies are potent inducers of cell death triggered by the apoptosis of cancer cells [20,21]. Recent advances in biology have led to a better understanding of the molecular events important in the pathogenesis of AD, suggesting a critical relationship between DNA repair and AD pathogenesis. In the present review, we summarize the function of prominent DNA repair molecules and in particular tumor suppressor gene products, p53 and BRCA1, as a viewpoint of DNA damage and therapeutic modulation in AD. These studies indicate significant roles for tumor suppressor molecules in controlling AD progression, which would essentially facilitate more effective treatments for a better prognosis of AD.

## 2. Relationship between AD and BRCA1 in the DNA Repair Pathway

**Figure 1:**
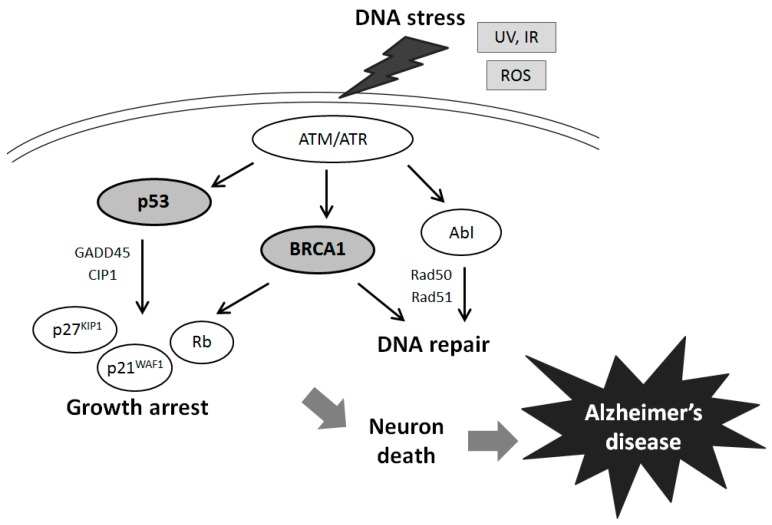
Schematic illustration of tumor suppressor signaling including BRCA1, p53, Rb, p21WAF1/p27KIP1, and ATM in Alzheimer’s disease. Examples of molecules involved in Alzheimer’s disease known to act on cell growth arrest and DNA repair via the regulatory pathways are presented. Note that some critical pathways have been omitted for clarity.

The tumor suppressor BRCA1 implicated in breast and ovarian cancers exerts various properties on the DNA repair system [20,22]. Actually, hereditary breast cancer with genomic aberration in BRCA1 is a type of cancer with defects in the DNA repair pathway [23]. In addition, mutations of a single allele of the BRCA1 gene are associated with increased genomic instability in breast epithelial cells [24,25], which further accelerate the mutation rate of other critical genes. Along with transcriptional activation and growth inhibition [26], BRCA1 is involved in transcription coupled in DNA repair of oxidative DNA damage [27], and regulatory roles for the G2/M cell cycle [28]. Furthermore, BRCA1 overexpression attenuates the production of ROS and up-regulating nitric oxide synthase [29]. ROS are formed resulting in oxidative DNA damage which is followed by increased DNA repair activity so that initial DNA damage is efficiently repaired. These well-known functions of BRCA may also be associated with AD (Figure 1). For example, oxidative DNA damage as well as RNA damage has been well documented in the aging brain, contributing to the development of AD [30]. Even the cases of slight cognitive impairment of the aging brain displays the same abnormalities, which provoke the search for DNA repair mechanisms in the case of neurodegeneration [31]. Evidence of the oxidative DNA damage profile in AD brings a speculation that BRCA1 may play an important role in AD pathogenesis [16]. BRCA1 is also known to have a role in maintaining telomere function and as such the presence of BRCA1 is indicative of DNA damage, both of which are pathogenic changes in neurodegeneration. Since the prevalence of BRCA1 increases as the disease progresses, transcription of BRCA1 may be activated early in the progression of neurodegeneration, suggesting that changes of DNA repair take place early in the progression of the disease [7,17].

**Figure 2:**
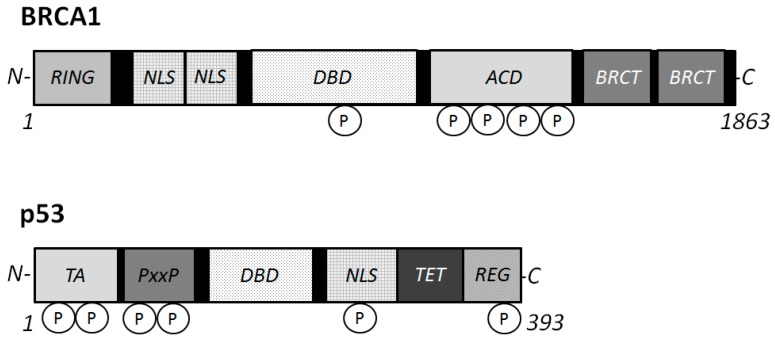
Schematic structures of BRCA1 and p53 proteins. The predicted consensual domain structures for each protein are depicted. The functionally important sites are also shown. Note that the sizes of protein are modified for clarity. RING = (Really Interesting New Gene) finger domain; TA = transactivation domain; PxxP = proline rich region; NLS = Nuclear Localization Signal; DBD = DNA binding domain; BRCT = BRCA1 *C*-terminus domain; REG = regulatory domain; TET = tetramerization domain.

The *BRCA1* gene is generally expressed in dividing neuronal cells during development [32], and encodes for a 1863 amino acids protein with an amino terminal zinc ring finger motif and two putative nuclear localization signals [33] (Figure 2). The amino-terminal domain retains E3 ubiquitin ligase activity [34], and the carboxyl-terminal domain is involved in association with specific phospho-proteins. The BRCA1 stimulates the CDK inhibitor p21 WAF1 and p53 tumor suppressor protein [35], which regulates several genes that control cell cycle checkpoints. The role of BRCA1 in the cell cycle control has been assumed by its ability to interact with various cyclins and cyclin-dependent kinases. In addition, BRCA1 has binding properties for BRCA2, Rb, Rad50 and Rad51 [36], in order to activate the cell cycle checkpoints. For example, BRCA1 is colocalized with Rad51, a DNA recombinase related to the bacterial RecA protein [37]. They may be involved in DNA double strand break repair. Rb is another tumor suppressor and a cell cycle checkpoint protein, which is also a potential diagnostic biomarker for AD [38]. The DNA repair Rad50 protein complexes are present in neurons of adult human cortex and cerebellum, but found significantly decreased in the neurons of AD brain cortex [39]. The presence of phosphorylated BRCA1 (Figure 2) has been characterized in a condition of DNA damage [40]. The BRCA1 protein becomes hyper-phosphorylated after exposure to DNA damaging agents and BRCA1 activity seems to be regulated by phosphorylation in response to DNA damage. Phosphorylated BRCA1 has also been implicated to play a role in maintaining genomic integrity in mitochondria [41]. Recent work has reported that BRCA1 functions in telomere maintenance, a distinctive feature of degenerating neurons in the AD brain [42].

## 3. Activation and Inactivation of the p53 Tumor Suppressor Involved in DNA Repair and AD

**Figure 3:**
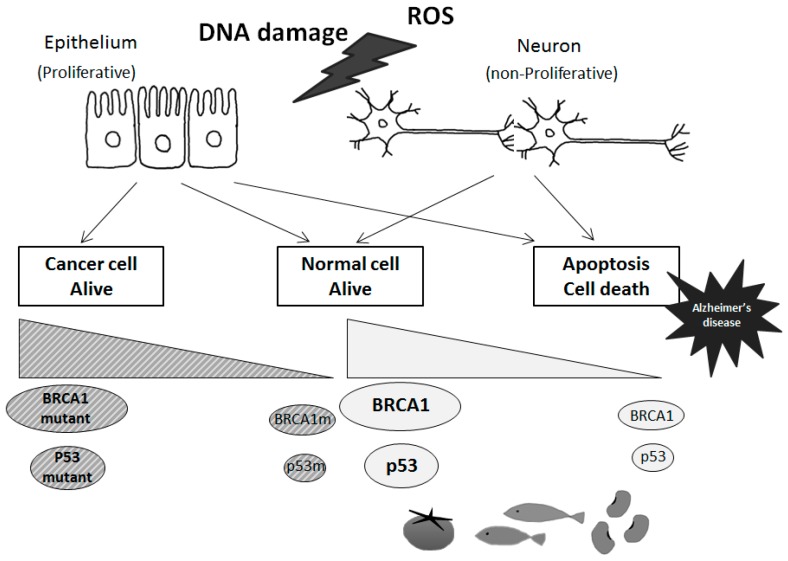
Tumor suppressor-dependent cellular fates in cancer and Alzheimer’s disease. Schematic illustrations of the tentative model for BRCA1 and p53 with these mutants are shown. In response to genotoxic signals, BRCA1 and p53 may be activated and induce cell cycle arrest. To maintain genomic stability, cell machinery would either induce DNA repair or activate apoptosis of damaged cells, which would then lead to a predisposition to Alzheimer’s disease in case of failure of the DNA repair. Some diets could stimulate neuronal tumor suppressor molecules expression and/or activities, which could also contribute to Alzheimer’s disease prevention.

Functional activities of p53 signaling may be involved in reverse associations between AD and cancer [43,44] (Figure 3). When p53 is activated in response to various types of cellular stresses including DNA damage, p53 induces cell cycle arrest among other functions, and failure of the DNA repair machinery leads to p53-mediated induction of apoptotic cell death. In order to maintain genomic stability, the decision is made whether to induce DNA repair or apoptosis of damaged cells. If the cell machinery were shifted to altered sensitivity for the response to stressors, the cells would be more prone to cell death, or to develop a cancer [44]. Because much of the response to genotoxic stress flows through the p53 pathway, p53 might be expected to play a key role in the cell consequences of genotoxic stress. In fact, the p53 protein is involved in many signaling pathways for the regulation of cell growth and apoptosis. It is suggested that cell cycle checkpoint abnormalities or loss of DNA damage protection in AD may occur via intensifying oxidative stresses [16,45]. Indeed, p53 and BRCA1 regulate a number of genes, and protect against genomic instability. In addition, a large number of molecules capable of activating p53 have been identified, and the effects are mediated by different downstream effectors and targets. Among them, a cyclin-dependent kinase (CDK) inhibitor and p21 WAF1 are key mediators of p53 action [46]. The p21 WAF1 inhibits cell cycle progression due to the interaction with cyclins and CDK complexes. Remarkably, it has been reported that mRNA levels of p21 WAF1 in AD is increased [47]. In addition, a pathogenic presenilin mutation in AD causes a specific increase in p53 and p21 protein level [48]. In order to promote cell death or cell survival, p53 and p21 may function depending on the type of stress stimuli, the cell type, and its protein activity [49,50]. By a stress such as hypoxia, p53 is also induced and activated in the nucleus [51,52]. Because p53 plays the most important role in the regulation of gene transcription, modification of p53 may be a key determinant of cell fate.

p53 is ubiquitously expressed in all cell types as an inactive transcription factor. However, p53 is frequently mutated in multiple cancer tissues indicating that p53 plays a critical role in preventing cancers. In general, mutant genes can be classified as a loss of function or a gain of function depending on the type of mutations. So, there are two types of p53 genes, the wild type p53 gene and the mutant p53 gene in diseases [53,54]. Oncogenic p53 mutations generally confer the mutant protein with a dominant-negative activity over the residual wild-type p53 gene product [53,54]. Many forms of mutant p53 acquire dominant-negative activities, and sometimes acquire oncogenic properties by themselves [53,54]. On the other hand, wild-type p53 activation with high protein expression may lead to regression of an early neoplastic lesion. These activities of p53 are also regulated by post-translational modification [55]. Phosphorylation (Figure 2) and acetylation, subcellular localization, and interaction with other cellular proteins are likely to influence the function of p53 [56]. Various cell proliferation- and apoptosis-signal transduction pathways may be constructed on complicated intra-cellular networks between oncogenes and tumor suppressor genes with their downstream factors. Thus, p53 regulates gene expression and plays an important role in modulation of signal transduction pathways. As mentioned above, p53 is involved in repair of damaged DNA and thus prevents accumulation of mutations, thereby suppressing tumor development. Accumulation of DNA stress and DNA damage beyond the capacity of p53 in neuronal cells may lead to neuronal apoptosis, and subsequently induce AD.

## 4. Some Diets Involved in Tumor Suppressor Expression May Contribute to the Neuro-Protection in AD

Possible therapeutic tactics may be achieved by surveillance required for maintaining cellular homeostasis. Therefore, it might be important and cost-effective to define appropriate plans to get benefits from life-style and/or diets to control the expression of tumor suppressors. Lycopene is a fat soluble red pigment carotenoid that significantly reduces the risk for prostate cancer. It is naturally occurring in many fruits and vegetables such as tomatoes. Treatment with lycopene increases BRCA1 gene expression in breast cancer cell line MCF-7 and HBL-100 [57]. Furthermore, treatment with lycopene increases phospho-BRCA1 in MCF-7 cells [58]. Manganese-induced perturbation in the antioxidant system with increase of acetylcholinesterase as demonstrated by AD is prevented by lycopene treatment [59], suggesting potential protective effects of lycopene against AD. Treatment with soy phytoestrogens may invert DNA hyper-methylation and return the gene expression of BRCA1 [60]. Actually, phytoestrogen-rich diets are able to increase the mRNA of BRCA1 [61]. The mRNA expression of BRCA1 is also found up-regulated in the mammary glands of rats exposed to genistein during pre-puberty [62]. Indole-3-carbinol and genistein are naturally occurring chemicals derived from green vegetables and soy, respectively. It has been shown that both indole-3-carbinol and genistein induce the gene expression of BRCA1 in MCF-7 and T47D breast cancer cells [63]. In addition, phytoestrogens have been shown to reduce AD related pathology, potentially alleviating risk of AD progression [64], which effectively attenuates oxidative damage and improves parameters related to aging and AD [65]. Furthermore, phytoestrogens such as genistein and daidzein may decrease DNA methylation in the BRCA1 genome [66,67]. On the contrary, however, it is also noted that consumption of soy food products may contribute to the increasing risk of Alzheimer’s dementia [68].

Ginsenoside, one of components in American ginseng herb, activates p53 [69], which could improve spatial learning and memory, suggesting a useful agent for preventing and treating cognitive impairment in AD [70]. It has been suggested that pretreatment of ginsennoside has neuroprotective effects in a rat model of AD [71]. In addition, thymoquinone, the most abundant component in black seed, is associated with a rise of p53 mRNA and the downstream p53 target genes [72]. It is suggested that thymoquinone has potential neuroprotective effects in rat hippocampal and cortical neurons and thus may be a promising candidate for AD prevention [73,74]. Treatment with *Magnolia officinalis* (*M. officinalis*) extract up-regulates expression of p21 WAF1 and p27 KIP1 [75]. In addition, ethanol extracts of *M. officinalis* effectively prevents memory impairment in a transgenic mouse model of AD [76]. Moreover, neuroprotective effects of honokiol and magnolol, compounds from *M. officinalis*, on beta-amyloid-induced neuronal toxicity have been reported [77]. Baicalin, an herb-derived flavonoid compound, enhances the expression of p27 KIP1 [78]. The effect of baicalin attenuates pathological changes and memory deficits such as in AD, suggesting that baicalin may be a potential candidate for the treatment of AD [79].

Several other promising herbs or life-style conditions involved in p53 activity are as follows. Treatment with thorns from a medicinal herb, *Gleditsia sinensis*, have been shown to increase cell cycle arrest during the G2/M phase, and is associated with increased p53 protein level [80]. Treatment with extract of *Gleditsia sinensis* thorns is also associated with up-regulation of p21 WAF1 [81]. In addition, mRNA levels both of p53 and of p21 WAF1 are increased with usage of Kanglaite, an extract from Coix seed. Kanglaite appears to extend the protein half-life of p53 [82]. On the other hand, treatment with ethyl acetate extract of *Saussurea involucrate* induces p21 WAF1 and p27 KIP1 expression, independent of the p53 pathway [83]. Zinc is an essential element that is integral to some transcription factors with zinc finger structure, which regulate key cellular functions such as responses to oxidative stress and DNA damage repair. Zinc is also involved in the stabilization and activation of p53 which appears to be an important component of the apoptotic process [84]. Signaling pathways of p53 with zinc donor/acceptor pair may be critically involved in life and death decisions of cells [85]. Zinc thus provides an effective dietary preventive approach to cancers and AD, and zinc could also be effective in the treatment of some cancers and AD [84,85]. Furthermore, zinc is now accepted as a potent neuromodulator, affecting a variety of signaling pathways at the synapse that are critical to cognition [86]. Hypoxia-induced p53-mediated signaling may well be effective in the targeting of hypoxic cells [87]. The DNA damage response is induced in cells by hypoxia. In addition, brain hypoxia may be associated with increased concentration of neurotoxins and cellular stresses in AD brains. The activity of p53 may be altered by toxic amyloid-beta in AD, as well as by hypoxia to trigger neural apoptotic cell death. Overall, promising alternatives to the use of medications against AD seem to be some diets with the use of food supplements to modulate the expression and/or activity of tumor suppressor molecules. It will be a challenge to seek out how to use these medicinal materials and/or conditions for the beneficial modulation of critical processes required for maintaining cellular homeostasis that are associated with a pathologic situation characterized by AD (Figure 3).

## 5. Perspective

To maintain normal cellular function, neurons can struggle to avoid oxidative damage throughout aging processes. If the cells cannot keep the balance, the excess toxic stresses may cause neuronal cell death. Because AD is a disease that could last more than ten years, neurons may attempt to survive by attempting to control the damaged or deregulated cells rather than succumbing to apoptosis immediately. These speculations raise the possibility that BRCA1 and p53 accumulate in neurons early stages of AD. In addition, the association of BRCA1 and p53 with neurodegenerative pathology in AD may implicate a neuro-protective function in healthy neurons in AD. Genomic instability as a feature in the pathogenesis of neurodegeneration in AD may as well be a feature of the DNA repair activity in survival neurons. Alteration of BRCA1 and p53 simultaneously and complexly indicate oncogenic and/or neurodegenerative stimuli which are found in cancer, AD, and other neuropathology. There are several examples showing cognitive improvements in dementia patients undergoing chemotherapy against cancer. Notably, tumor suppressors are crucial molecules at this point. Neuroprotective factors have been suggested as a possible target for drug design efforts with the goal of delaying neurodegeneration from neuronal apoptosis. This may represent the rational basis for the development of dietary treatment of AD. Further mechanistic studies are required to understand the precise molecular mechanisms, and to determine whether an appropriate dietary intake is related to the improved brain function and the preservation of brain health. Long-term clinical studies are also necessary to clarify effects of treatment in the management of AD.

## 6. Conclusions

The functions of BRCA1 and p53 are involved in the pathogenesis of AD as well as carcinogenesis. Some diets may contribute to neuro-protection, disease prevention, and possible better prognosis in AD via regulation of the expression of tumor suppressors.

## Abbreviation

AD: Alzheimer’s disease
ATM: ataxia telangiectasia-mutated
BRCA1: breast cancer gene 1
CDK: cyclin-dependent kinase
CDK2: cyclin-dependent kinase 2
DSBs: DNA double strand breaks
ROS: reactive oxygen species
